# Vitamin D receptor regulates intestinal proteins involved in cell proliferation, migration and stress response

**DOI:** 10.1186/1476-511X-13-51

**Published:** 2014-03-19

**Authors:** Hagen Kühne, Alexandra Schutkowski, Susann Weinholz, Christina Cordes, Angelika Schierhorn, Kristin Schulz, Bettina König, Gabriele I Stangl

**Affiliations:** 1 Institute of Agricultural and Nutritional Sciences, Martin Luther University Halle-Wittenberg, Von-Danckelmann-Platz 2, D-06120 Halle (Saale), Germany; 2 Department of Applied Biosciences and Process Engineering, Anhalt University of Applied Sciences, D-06366 Köthen, Germany; 3 Institute for Biochemistry and Biotechnology, Martin Luther University Halle-Wittenberg, D-06120 Halle (Saale), Germany; 4 Institute of Medical Immunology, Martin Luther University Halle-Wittenberg, D-06112 Halle (Saale), Germany

**Keywords:** VDR-deficiency, Small intestine, Proteomics, Laminin receptor, Cell adhesion, Mice, Stress response

## Abstract

**Background:**

Genome-wide association studies found low plasma levels of 25-hydroxyvitamin D and vitamin D receptor (VDR) polymorphisms associated with a higher prevalence of pathological changes in the intestine such as chronic inflammatory bowel diseases.

**Methods:**

In this study, a proteomic approach was applied to understand the overall physiological importance of vitamin D in the small intestine, beyond its function in calcium and phosphate absorption.

**Results:**

In total, 569 protein spots could be detected by two-dimensional-difference in-gel electrophoresis (2D-DIGE), and 82 proteins were considered as differentially regulated in the intestinal mucosa of VDR-deficient mice compared to that of wildtype (WT) mice. Fourteen clearly detectable proteins were identified by MS/MS and further analyzed by western blot and/or real-time RT-PCR. The differentially expressed proteins are functionally involved in cell proliferation, cell adhesion and cell migration, stress response and lipid transport. Mice lacking VDR revealed higher levels of intestinal proteins associated with proliferation and migration such as the 37/67 kDa laminin receptor, collagen type VI (alpha 1 chain), keratin-19, tropomyosin-3, adseverin and higher levels of proteins involved in protein trafficking and stress response than WT mice. In contrast, proteins that are involved in transport of bile and fatty acids were down-regulated in small intestine of mice lacking VDR compared to WT mice. However, plasma and liver concentrations of cholesterol and triglycerides were not different between the two groups of mice.

**Conclusion:**

Collectively, these data imply VDR as an important factor for controlling cell proliferation, migration and stress response in the small intestine.

## Background

Vitamin D is well known for its role in regulation of calcium homeostasis and bone metabolism [1], but it also exerts numerous other non-skeletal biological effects in almost all tissues [2]. It is currently assumed that vitamin D participates in the regulation of up to 5% of the human genome [3]. One important target tissue of vitamin D is the intestine where it regulates calcium and phosphate absorption [4-6]. Non-skeletal vitamin D effects in the intestine have been characterized mainly in the colon and include the regulation of tight junction proteins in the epithelial layer [7]. Vitamin D further modulates colonic T-cell responses and inflammation processes [5,8-10], and regulates the proliferation of intestinal cells in the colon [11-13]. It should therefore be presumed that vitamin D deficiency leads to mucosal dysregulation. The observed associations of low vitamin D levels or VDR deficiency with inflammatory bowel diseases in epidemiologic studies [14-17], genome-wide association studies [18] and experimental murine models [7,19-21], and the linkage between vitamin D deficiency and colorectal cancer [22,23] may reflect the proposed intestinal dysregulation in response to vitamin D deficiency. Apart from the vitamin D function in mineral absorption, there is a gap of knowledge on the role of vitamin D in the small intestine, although the cells from the small intestine reveal a higher VDR expression than most of the other tissues [24]. The VDR is a nuclear receptor that mediates the cellular effects of vitamin D by binding to vitamin D response elements of target genes [25]. Therefore, mice lacking VDR are an appropriate model to elucidate the overall functions of vitamin D in selected tissues [26]. To target the gap of knowledge on vitamin D function in the small intestine, we performed quantitative comparative protein expression profiling of the small intestinal mucosa of VDR knockout (KO) versus wildtype (WT) mice by applying two-dimensional-difference in-gel electrophoresis (2D-DIGE) in combination with ESI-QTOF-MS/MS.

## Results

### Analysis and identification of differentially expressed proteins

**Figure 1:**
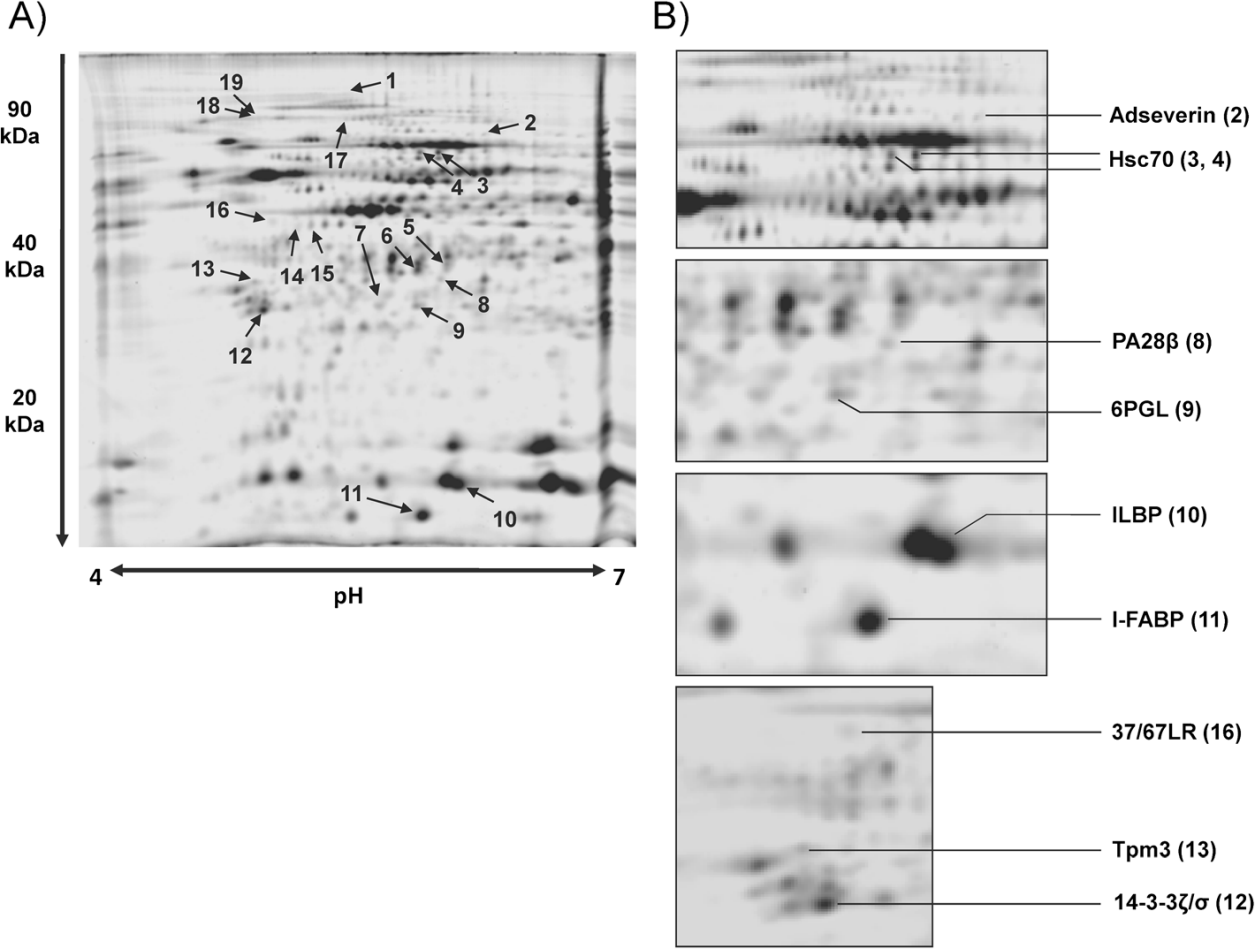
**2D spot pattern of analytical gels. A)** Gels are composed of small intestinal mucosa of vitamin D receptor knockout (VDR-KO) and corresponding wildtype (WT) mice. In total, 15 μg of fluorescence labeled proteins were subjected to 2D-DIGE. **B)** Enlarged sections showing selected spots of the analytical gel in A). For spot description, see Table 1.

**Figure 2:**
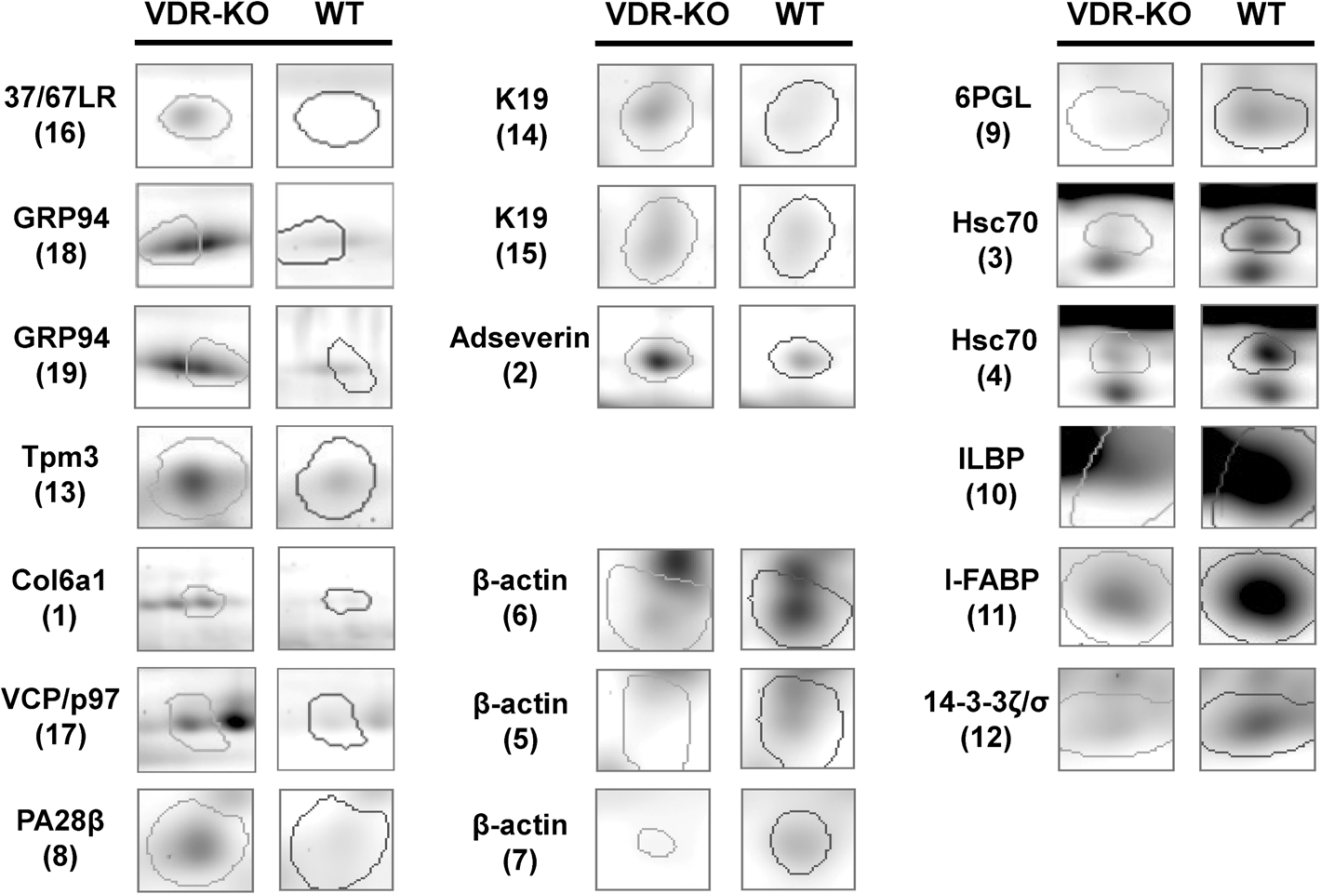
**Representative spot comparison of differentially expressed proteins of vitamin D receptor knockout and wildtype mice.** Regulated and identified proteins from small intestinal mucosa of vitamin D receptor knockout (VDR-KO, left) mice are pictured in comparison to their respective wildtype (WT) mice (right) counterpart. Spot number is given in brackets.

In order to determine the differential protein expression pattern of the small intestinal mucosa of VDR-KO and WT mice, protein extracts were analyzed by 2D-DIGE. In total, 569 protein spots could be detected on the resulting consensus 2D gel. Comparative analysis of the protein expression profiles revealed that 82 protein spots can be classified differentially expressed (by a regulation factor > 2.0 (up- or down-regulated)) between VDR-KO and corresponding WT mice. 19 spots could be assigned to 14 unique proteins by MS/MS identification (Table 1). A representative 2D gel image is shown in Figure 1 and direct spot comparisons are shown in Figure 2. Three spots were identified as β-actin with various pIs and molecular weights. Also, 94 kDa glucose-regulated protein (GRP94), heat shock cognate 71 kDa protein (Hsc70) and keratin-19 (K19) were detected in multiple spots which may represent post-translationally modified forms of these proteins. Proteins that were up-regulated in small intestinal mucosa of VDR-KO compared to WT mice included proteins that are involved in cell adhesion (37/67 kDa laminin receptor (37/67LR), collagen type VI (alpha 1 chain) (Col6a1)), cytoskeleton proteins (tropomyosin-3 (Tpm3), adseverin, K19), stress response and protein trafficking proteins (GRP94, valosin-containing protein (VCP/p97)) and the proteasome activator subunit 2 (PA28β). Proteins that were down-regulated in VDR-KO compared to WT mice are two lipid transport proteins (ileal lipid-binding protein (ILBP), intestinal fatty acid-binding protein (I-FABP)), the cytoskeleton component β-actin, the 14-3-3 protein isoform ζ/δ, the heat shock cognate protein Hsc70 and 6-phosphogluconolactonase (6PGL). Note, PA28β and 6PGL failed to overcome the Mascot significance level of 56 and may therefore be indefinite despite adequate sequence coverage.

**Table 1:** Differentially expressed proteins and mRNA abundances of genes in the small intestinal mucosa of VDR-KO relative to WT mice

**UniProtAcc.**	**Protein**	**Spot no.**	**Fold change**^**a**^	**No. of peptides**	**Coverage (%)**	**pI (theor.)**	**Mascot score**	**mRNA fold change**^**a**^	**Function**
P14206	37/67 kDa laminin receptor (37/67LR)	16	+10.8	9	39	4.80	422	n.s.	Membrane receptor for laminin
P08113	94 kDa glucose-regulated protein (GRP94)	18, 19	+5.0, +5.5	22	28	4.74	307	-1.4*	Chaperone, stress response
P21107	Tropomyosin 3 (Tpm3)	13	+4.3	11	28	4.68	225	n.d.	Cytoskeleton
Q04857	Collagen type VI (alpha 1 chain) (Col6a1)	1	+4.2	9	10	5.20	88	+1.8*	Extracellular matrix protein
Q01853	Valosin-containing protein(VCP/p97)	17	+3.8	18	28	5.14	204	n.s.	Chaperon, stress response, protein degradation
P97372	Proteasome activator complex subunit 2 (PA28β)	8	+3.1	8	40	5.54	35^c^	-1.6*	Proteasome activation
P19001	Keratin, type I cytoskeletal 19 (K19)	14, 15	+2.1, +2.9	14	39	5.28	127	n.s.	Cytoskeleton
Q60604	Adseverin	2	+2.8	18	30	5.46	88	n.s.	Cytoskeleton
P60710	β-actin	5, 6, 7	-6.5, -4.2-4.1	5	15-21	5.29^b^	54-70	-2.0*	Cytoskeleton
Q9CQ60	6-phosphogluconolactonase (6PGL)	9	-2.7	3	15	5.55	39^c^	n.s.	Carbohydrate metabolism
P63017	Heat shock cognate 71 kDa protein (Hsc70)	3, 4	-2.5, -2.6	31	52	5.37	586	n.s.	Chaperone, stress response
P51162	Ileal lipid-binding protein (ILBP)	10	-2.5	8	68	5.91	210	n.d.	Lipid metabolism
P55050	Fatty acid-binding protein 2 (I-FABP)	11	-2.4	4	31	6.62	102	n.s.	Lipid metabolism
P63101	14-3-3 protein ζ/δ	12	-2.3	18	63	4.73	353	-1.6*	Signal transduction

^a)^“+” indicates up- and “–“ down-regulation in VDR-KO vs. WT mice; ^b)^full length protein; ^c)^below Mascot significance threshold of 56; *^)^Significantly different from WT mice (p < 0.05, Student’s *t*-test); n.s. non-significant; n.d. not determined.

### Verification of 2D-DIGE data by western blotting and RT-PCR

**Figure 3:**
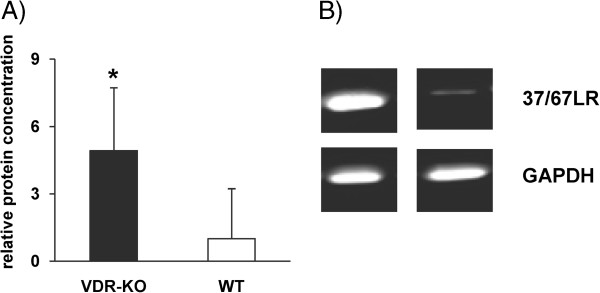
**Western blot analysis of 37/67 kDa laminin receptor. A)** Protein expression of 37/67 kDa laminin receptor (37/67LR) of small intestinal mucosa samples of vitamin D receptor knockout (VDR-KO) and wildtype (WT) mice are represented as means ± s.d. (n = 6). *Significantly different from WT mice (p < 0.05, Student’s t-test). **B)** Representative immunoblot of 37/67LR and GAPDH from small intestinal mucosa samples of VDR-KO and WT mice.

To verify the 2D-DIGE results, the 37/67LR protein expression was analyzed by western blot analysis. 37/67LR was chosen because of its tenfold higher expression in VDR-KO compared to WT mice. Western blot analysis revealed that protein expression of 37/67LR was about fivefold higher in the small intestinal mucosa of VDR-KO than in the mucosa of WT mice (Figure 3), thus indeed supporting the initial proteomic profiling results.

**Figure 4:**
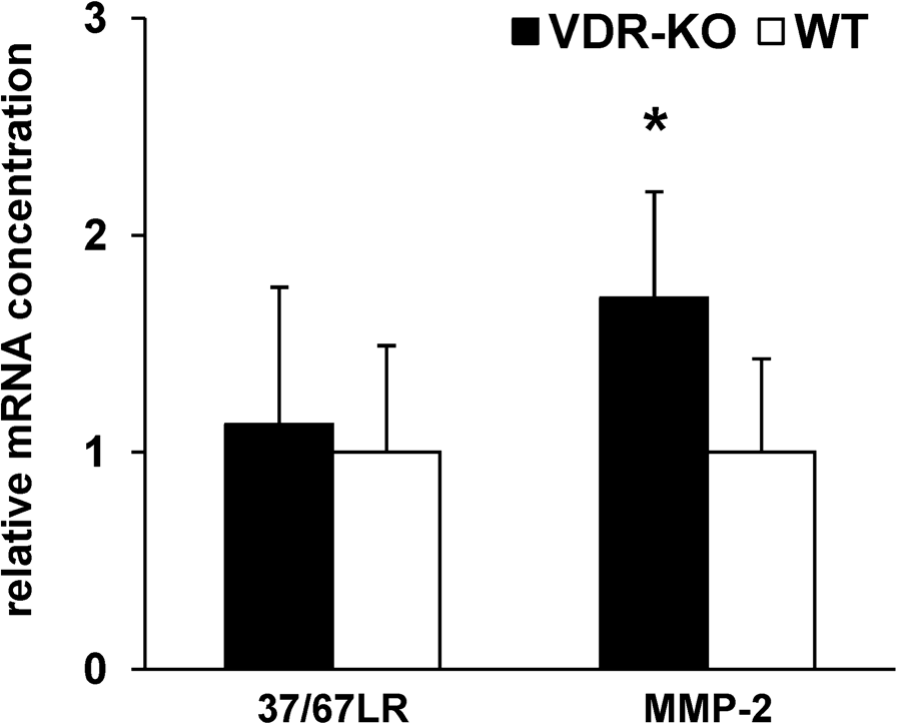
**Relative mRNA levels of 37/67 kDa laminin receptor (37/67LR) and matrix-metalloproteinase-(MMP)-2.** Relative mRNA levels in small intestinal mucosa of vitamin D receptor knockout (VDR-KO) and wildtype (WT) mice were determined by real-time detection RT-PCR analysis. Expression values were normalized to HPRT and Ppia. Bars represent means ± s.d. (n = 6). *Significantly different from WT mice (p < 0.05, Student’s t-test).

To analyze whether the observed differences in protein expression between the VDR-KO and the WT mice were linked to changes in the mRNA levels, we determined the relative mRNA concentrations of the corresponding genes. Here we show that the relative mRNA abundances of β-actin, Col6a1 and 14-3-3 protein ζ/δ were in line with protein expression data (Table 1). The intestinal mRNA abundances of GRP94 and PA28β which were slightly lower in the VDR-KO mice than in the WT mice completely differed from the protein data, and the mRNA abundances of seven other genes were not different between VDR-KO and WT mice, assuming post-transcriptional effects which are responsible for the discrepancy between mRNA and protein data (Table 1). As indicated in Figure 4, no difference in relative mRNA concentration of 37/67LR was found in small intestinal mucosa of VDR-KO and WT mice. Increased expression of the membrane form of 37/67LR is associated with increased mRNA expression and activity of matrix metalloproteinase (MMP)-2 [27]. Therefore, we also analyzed the mRNA abundance of MMP-2. Data reveal that mRNA-concentration of MMP-2 was higher in the small intestine of VDR-KO mice than in small intestine of WT mice (1.7-fold; p < 0.01; Figure 4).

### Lipid concentrations in plasma and liver

Triglyceride and cholesterol concentrations of plasma and liver did not differ between VDR-KO and WT mice (triglycerides in plasma: VDR-KO, 1.85 ± 0.31 mmol/l, WT, 1.71 ± 0.48 mmol/l; liver triglycerides: VDR-KO, 60.3 ± 8.4 μmol/g, WT, 57.9 ± 8.8 μmol/g; cholesterol in plasma: VDR-KO, 3.34 ± 0.28 mmol/l, WT, 3.39 ± 0.54 mmol/l; liver cholesterol: VDR-KO, 10.9 ± 1.4 μmol/g, WT, 12.4 ± 2.4 μmol/g).

## Discussion

This study aimed to investigate the role of the vitamin D on small intestinal mucosa proteins by use of VDR-KO mice and 2D-DIGE analysis. Here we could identify 14 proteins that were differently expressed in VDR-KO and WT mice indicating a direct or indirect involvement of VDR in regulation of these proteins. The identified proteins are involved in cell proliferation, cell migration and stress response.

Intestinal integrity is governed by a variety of signaling pathways that balance cell proliferation and differentiation. Under physiological conditions, cell replenishment is characterized by continuous proliferation of crypt epithelial cells, migration of the cells along the crypt-villus axis and extrusion at the villus tip. Cell migration requires cycles of polarized attachment of cells to the underlying matrix, and the establishment of the polarized state. Regulated re-arrangements of the cytoskeleton and coordinated intracellular trafficking of organelles and membrane proteins are important steps in these processes. Here, we found that VDR deficiency is associated with the up-regulation of proteins that are involved in processes of proliferation and migration of epithelial cells in the small intestine. These include 37/67LR, Col6a1, Tpm3, adseverin, K19 and GRP94. Among those proteins, the strongest change was observed for 37/67LR, which functions in its membrane bound form as a non-integrin receptor for the basement membrane glycoprotein laminin [28,29]. 37/67LR is over-expressed in a variety of common cancers and its expression level correlates with aggressiveness and metastatic potential [29]. Recent data demonstrate that 37/67LR predominantly appears in the undifferentiated/proliferative region of the human intestinal crypt and regulates adhesion and proliferation of epithelial cells [30]. We assume that the strong expression of 37/67LR in the VDR-KO mice might reflect a disturbed balance between cell proliferation and differentiation in favor of proliferation. This confirms the anti-proliferative function of vitamin D and VDR which has been described for the colon and colon carcinoma cells [31,32]. The finding that the mRNA expression of MMP-2 was also enhanced corroborates the high abundance of 37/67LR in the small intestine of mice lacking VDR [27]. However, increased protein expression of 37/67LR was not associated with increased mRNA levels indicating posttranscriptional regulation. A second protein that was up-regulated in the VDR-KO mice was Col6a1. Collagen VI functions as a *bona fide* basal lamina component in the intestine, and is involved in epithelial cell behavior and cell-fibronectin interactions [33].

Besides cellular adhesion components, also cytoskeletal components were up-regulated in the intestine of VDR-KO mice. K19, that belongs to one of those up-regulated cytoskeletal proteins, functions as an intermediate filament mainly in proliferating crypt cells [34] and is associated with a less differentiated phenotype of Caco-2-cells [35]. Tpm3 and the gelsolin-family member adseverin are two other differentially expressed proteins which are involved in actin-filament assembly and turnover [36,37]. In the context of an altered expression of β-actin in the small intestine of VDR-KO mice, the findings assume changes in the regulation of actin-filament dynamics in these animals, and confirm other studies that found vitamin D involved in modulation of cytoskeletal protein expression in e.g. muscle cells [38] and cell lines [39,40].

Vitamin D is supposed to be involved in reducing oxidative cell and endoplasmic reticulum (ER) stress [41-43], and colon cells of VDR-KO mice show higher levels of biomarkers for oxidative stress than those of WT mice [32]. The current study found a couple of differentially expressed stress response proteins in the small intestine of VDR-KO mice compared to WT mice that may underline the vitamin D function in stress response. In particular, we observed alterations in the intestinal expression of GRP94, a member of the HSP90 family, the ATP-driven VCP/p97, Hsc70, a molecular chaperone of the HSP70 family, and the proteasome activator subunit PA28β in mice lacking VDR compared to WT mice. These proteins are involved in adaptive mechanisms to stress response, especially in processing misfolded proteins (ubiquitination, refolding) [44-47]. Since the small intestine is normally exposed to a multitude of stressors like harmful nutritional components, microorganisms, toxins and luminal antigens, it can be speculated that vitamin D may modify the response to cells to those stressors.

The multifunctional protein 14-3-3ζ/δ was less expressed in small intestine of VDR-KO than of WT mice. Proteins of the 14-3-3 family are highly conserved eukaryotic proteins that regulate many cellular processes by altering the conformation, activity or subcellular localization of target proteins, e.g. cytoskeletal components [48-50]. Interestingly, 14-3-3ζ/δ is also involved in regulation of Wnt/β-catenin signaling in intestinal stem cells, a pathway that is implicated in intestinal development and regeneration [51], and that has been associated with the differentiation-promoting effect of vitamin D/VDR in colon carcinoma cells [23,52].

Besides alterations in proteins involved in cell proliferation, cell migration, cytoskeletal organization and stress response, the small intestine of VDR-KO mice showed reduced expression of ILBP and I-FABP, proteins that are involved in lipid metabolism and transport. Recent findings show an enlarged size of total bile acid pool, a stimulated bile acid synthesis and a reduced gene expression of bile acid transporters in the liver but not in intestine of VDR-KO compared to WT mice [53]. In that study, analyzed mRNA expression levels of ILBP show no differences between VDR-KO and WT mice which is confirmed by our results. Nevertheless, our study shows reduced protein expression of ILBP in VDR-KO mice indicating the involvement of post transcriptional mechanisms in regulation of ILBP. Expression of ILBP is stimulated by bile acid concentration [54], thus local differences in bile acid concentration may account for reduced ILBP protein expression in intestine of VDR-KO mice. FABP is usually involved in the transport of long chain fatty acids into enterocytes [55]. Despite the observed reduction in protein levels of FABP in the VDR-KO mice compared to the WT mice, we found no differences in plasma and liver lipids between these two groups of mice. These data confirm recently published data that show comparable fatty acid absorption rates between VDR-KO and WT mice [56]. We therefore assume that the diminished expression of both transporters may not induce obvious disturbances in lipid metabolism.

## Conclusions

Collectively, these data imply that the small intestine of mice lacking VDR expresses higher levels of proteins that are principally involved in cell proliferation, cell migration and stress response than corresponding WT mice. Thus, we can conclude that vitamin D may play a direct or an indirect role in balancing cell proliferation, cell migration and stress response in the small intestine.

## Methods

### Animals and study design

Six 12 to 15-week-old male vitamin D receptor knockout mice (VDR-KO; B6.129S4-VDR^tm1Mbd^>/J; Jackson Laboratory, Bar Habor, USA) and six age-matched male wildtype (WT) mice (C57BL/6 J, Charles River, Sulzfeld, Germany) were fed a rescue diet containing 2% calcium and 1.25% phosphorus. Other basal components of the diet were (in g/kg) casein (200), sucrose (200), lactose (200), starch (49.5), coconut fat (200), soybean oil (10), cholesterol (1.5), cellulose (50), DL-methionine (2), vitamin and mineral mixture (87), containing 1,000 IU vitamin D_3_. All mice were kept individually in a room controlled for temperature (22 ± 2°C), relative humidity (50–60%) and a 12-h light, 12-h dark cycle. All mice were allowed free access to food and water. The experimental procedures described followed established guidelines for the care and handling of laboratory animals and were approved by the council of Saxony-Anhalt (approval number: 42502-5-34 MLU).

### Sample collection

Prior to killing by decapitation under light anesthesia with diethyl ether all mice were food deprived for 10 hours. Blood was collected into EDTA tubes. Plasma was obtained by centrifugation at 1,500 *g* for 20 min and stored at -20°C. The small intestine (from pylorus to ileocecal valve) was completely excised and washed several times with cold NaCl solution (0.9%). Intestinal mucosa was harvested by scraping the surface of the small intestine. Obtained mucosa samples were snap-frozen in liquid nitrogen and stored at -80°C. The liver was excised and samples for lipid extraction were snap-frozen and stored at -80°C as well.

### Protein extraction

For isoelectric focusing (IEF), protein extracts of small intestinal mucosa of each mouse were prepared. Therefore, 20 mg of frozen small intestinal mucosa were mechanically disrupted (MPI FastPrep®24-System, MP Biomedicals LLC, Illkirch Cedex, France) in 200 μl of 50 mM Tris–HCl buffer (pH 7.5) containing protease inhibitor cocktail (1:100, Roche, Mannheim Germany). Crude homogenates were centrifuged for 15 min (16,000 *g*, 4°C), the supernatants were collected and protein concentrations were determined according to Bradford [57]. For subsequent fluorescence labeling and IEF, the samples were pooled group-wise at equal protein amounts and the resulting protein solutions were cleaned using the ReadyPrep™ 2D Cleanup Kit (Bio-Rad, Munich, Germany) according to the manufacturer’s protocol. The resulting protein precipitate was resuspended in a 2D compatible buffer (7 M Urea, 2 M thiourea, 4% CHAPS, 30 mM Tris–HCl, pH 8.5). Protein concentrations of the resuspended solutions were measured in dilution to discriminate urea interferences.

### 2D-DIGE analysis

Protein pools were labeled with fluorescence dyes using the Refraction-2D Kit (NH DyeAGNOSTICS GmbH, Halle (Saale), Germany) according to the manufacturer’s instructions. The internal standard consisted of homoequivalent amounts of protein from VDR-KO and WT mice. For analytical gels, 5 μg of labeled protein per animal group along with 5 μg of the internal standard were pooled and mixed with DeStreak rehydration buffer (GE Healthcare, Munich, Germany) containing 0.5% carrier ampholytes (pH 4–7, SERVA Electrophoresis, Heidelberg, Germany) and added to immobilized pH gradient strips (pH 4–7, 7 cm, linear, Bio-Rad, Munich, Germany) for passive sample loading overnight at room temperature. Preparative gels were loaded with 300 μg of total protein that was spiked with labeled protein for the subsequent matching process with analytical gels.

First dimension IEF was run on a Protean IEF Cell (Bio-Rad, Munich, Germany) followed by a two step equilibration process using equilibration buffer (50 mM Tris–HCl (pH 8.8), 6 M Urea, 2% SDS, 30% glycerol, bromophenol blue) supplemented with 2% DTT (step 1) or 2.5% iodoacetamide (step 2), and incubating the stripes for 15 min, respectively. Thereafter, proteins were separated using linear SDS-PAGE (12.5%) and fixed (50% ethanol, 10% acetic acid) for 1 h. The samples were processed in six replicates. For preparative gels, the fixation step was omitted and the fluorescence signal was recorded directly before staining with colloidal Coomassie blue [58]. Fluorescence signal acquisition was achieved using a Typhoon Trio laser scanner (GE Healthcare, Munich, Germany). Gel analysis was performed with the Delta2D software (Decodon, Greifswald, Germany). Protein spots that showed a regulation factor of at least 2 between the two groups were considered for further analysis.

### Protein identification by ESI-QTOF-MS/MS-analysis

Protein spots were excised from Coomassie-stained gels, washed, and digested with trypsin (Promega, Mannheim, Germany) in 10 μl of 10 mM ammonium bicarbonate (pH 8.0) overnight at 37°C. Peptides were extracted from gel pieces and injected into a nanoACQUITY UPLC system (Waters Co., Eschborn, Germany). 2 μl were injected via “microliter pickup” mode and desalted on-line through a symmetry C18 180 μm × 20 mm precolumn. The peptides were separated on a 100 μm × 100 mm analytical RP column (1.7 μm BEH 130 C18, Waters Co., Eschborn, Germany) using a typical UPLC gradient from 3.0 to 33.0% over 15 min and a flow rate of 600 nl/min. The mobile phases used were 0.1% formic acid in water and 0.1% formic acid in acetonitrile. The column was connected to a SYNAPT® G2 HDMS-mass spectrometer (Waters Co, Eschborn, Germany). The data were acquired in LC/MS^E^ mode switching between low and elevated energy on alternate scans. Subsequent correlation of precursor and product ions can then be achieved using both retention and drift time alignment. Using ProteinLynx Global SERVER 2.5.2 data were processed and searched against the SwissProt database specified for *Mus musculus* using Mascot search engine of Matrix Science [59].

### Western blot analysis

Standard western blot procedure was performed as described earlier [60]. Small intestinal mucosa samples were prepared as described above and 30 μg of protein of each individual mouse were resolved by electrophoresis in 12% SDS-PAGE gels. Membranes were incubated with anti-67 kDa laminin receptor antibody (1:1000; Abcam, Cambridge, UK; ab133645) and anti-GAPDH antibody (1:1000; Cell Signaling, Boston MA, USA; #5174S) respectively and subsequently detected with secondary HRP-conjugated antibody (1:1000; anti-rabbit IgG, Cell Signaling) using ECL Prime western blotting detection reagent (GE Healthcare, Munich, Germany).

### Real-time detection RT-PCR analysis

Total RNA was isolated from aliquots of mice small intestinal mucosa by peqGold Trifast™ reagent (Peqlab, Erlangen, Germany) according to the manufacturer’s protocol. cDNA synthesis and determination of mRNA abundance by real-time detection PCR (Rotor-Gene 6000, Corbett Research, Mortlake, Australia) were performed as described previously [61]. Calculation of the relative mRNA concentrations was performed according to [62]. PCR data were normalized to the reference genes hypoxanthine guanine phosphoribosyl transferase (HPRT) and peptidylprolyl isomerase A (Ppia). The following target-specific primers were used for real-time PCR analysis: peptidylprolyl isomerase A (NM_008907, Ppia), for, 5′-GTGGTCTTTGGGAAGGTGAA-3′, rev, 5′-TTACAGGACATTGCGAGCAG-3′; 37/67 kDa laminin receptor (NM_011029, Rpsa), for, 5′-CTTGACGTCCTGCAGATGAA-3′, rev, 5′-GGATTCTCGATGGCAACAAT-3′ and matrix metalloproteinase (MMP)-2 (NM_008610.2, Mmp2), for, 5′-GAGATCTTCTTCTTCAAGGAC-3′, rev, 5′-AATAGACCCAGTACTCATTCC-3′, β-actin (NM_007393, Actb), for, 5′- ACGGCCAGGTCATCACTATTG -3′, rev, 5′- CACAGGATTCCATACCCAAGAAG -3′. All other primer pairs were purchased from Sigma-Aldrich (http://www.kicqstart-primers-sigmaaldrich.com).

### Lipid analysis

Liver lipids were extracted using a mixture of n-hexane and isopropanol (3:2, v:v) [63]. Aliquots of lipid extracts were dried and dissolved in a small volume of Triton X-100 [64]. Concentrations of total cholesterol and triglycerides were determined as described previously [65].

### Statistical analysis

Data are presented as means ± standard deviation (s.d.). Means of VDR-KO and WT mice were compared by Student’s *t*-test using the Minitab Statistical software, version 13 (Minitab, State College, USA). Means were considered significantly different at p < 0.05.

## Abbreviations

2D-DIGE: Two-dimensional-difference in-gel electrophoresis; 6PGL: 6-Phosphogluconolactonase; Col6a1: Collagen type VI (alpha 1 chain); ESI-QTOF: Electrospray ionization-time of flight; GAPDH: Glyceraldehyde 3-phosphate dehydrogenase; GRP94: 94 kDa glucose-regulated protein; HPRT: Hypoxanthine guanine phosphoribosyl transferase; HRP: Horseradish peroxidase; Hsc70: Heat shock cognate 71 kDa protein; HSP: Heat shock protein; IEF: Isoelectric focusing; I-FABP: Intestinal fatty acid-binding protein; ILBP: Ileal lipid-binding protein; K19: Keratin-19; KO: Knockout; LR: Laminin receptor; MMP-2: Matrixmetalloproteinase-2; PA28β: Proteasome activator subunit 2; pI: Isoelectric point; Ppia: Peptidylprolyl isomerase A; Tpm3: Tropomyosin-3; UPLC: Ultra-performance liquid Chromatography, VCP/p97, Valosin-containing protein; VDR: Vitamin D receptor; WT: Wildtype.

## Competing interests

The authors declare that they have no competing interests.

## Authors’ contributions

HK, AlS and GS designed the experiment. HK, SW, CC, and KS carried out 2D experiments and expression analyses. AnS. delivered MS/MS data. HK, BK, AlS and GS carried out data analysis and interpretation. HK, AlS, BK, and GS wrote the manuscript. All authors read and approved the final manuscript.
